# The experimental data of investigating the efficiency of zinc oxide nanoparticles technology under ultraviolet radiation (UV/ZnO) to remove Acid – 32 – Cyanine 5R from aqueous solutions

**DOI:** 10.1016/j.dib.2018.10.037

**Published:** 2018-10-17

**Authors:** Mohammad Hadi Dehghani, Parvin Mahdavi, Zoha Heidarinejad

**Affiliations:** a Department of Environmental Health Engineering, School of Public Health, Tehran University of Medical Sciences, Tehran, Iran; b Institute for Environmental Research, Center for Solid Waste Research, Tehran University of Medical Sciences, Tehran, Iran; c Department of Environmental Health Engineering, Faculty of Health, Hormozgan University of Medical Sciences, Bandar Abbas, Iran; d Food Health Research Center, Hormozgan University of Medical Sciences, Bandar Abbas, Iran

**Keywords:** Acid – 32 – Cyanine 5R, Zinc oxide nanoparticles, Ultraviolet radiation, Aqueous solutions

## Abstract

The aim of this data was to evaluate the efficiency of zinc oxide nanoparticles plus ultraviolet radiation (UV/ZnO) technology to remove Acid – 32 – Cyanine 5R. The effect of optimal parameters including initial pH (5,10), contact time (2–20 min), initial dye concentration (0.5–2 mg/L), and zinc oxide dosage (0.1 and 0.2 g/L) was investigated. The data showed that under alkaline conditions (pH = 10) and 0.2 g/L of ZnO nanoparticles, the maximum dye removal efficiency was observed under UV/ZnO process conditions. Furthermore, with the increase in Acid – 32 – Cyanine 5R dye concentration, the removal efficiency of the dye diminished, while with prolongation of the radiation time, the removal efficiency increased. In the presence of ultraviolet radiation, there is a need to longer time and higher radiation intensity for complete removal of the dye. However, in the presence of ZnO nanoparticles alone, around 5–10% of the dye is removed. The highest removal efficiency of Acid – 32 dye was through radiation by ultraviolet lamp 150 W at an initial dye concentration of 1 mg/L, in pH 5 and 10, respectively, at 98.5% and 99% respectively. On the other hand, through hybrid use of UV/ZnO, within a shorter time, complete removal (100%) is achieved. Generally, use of UV/ZnO process can be utilized as a suitable method for dye wastewaters treatment.

## Specifications table

**Table:** 

Subject area	Environmental Chemistry
More specific subject area	Photo-catalytic removal
Type of data	Table and figure
How data was acquired	– Batch experiments were performed to collect the data of the influence of ZnO dosage, initial dye concentration, contact time and pH on Acid – 32 – Cyanine 5R dye removal. – The Acid – 32 – Cyanine 5R dye concentration was measured by a spectrophotometer device (Perkin – Elmer Lambada 25 – UV/Vis) at the wavelength of 400–700 nm.
Data format	Raw, Analyzed
Experimental factors	The data of effects of main experimental parameters including ZnO dosage, initial dye concentration, contact time and solution pH were acquired.
Experimental features	The objective of this data was to Acid – 32 – Cyanine 5R dye removal from aqueous solutions using i) nanophotocatalytic UV/ZnO, ii) radiation alone (UV), and iii) ZnO nanoparticles alone.
Data source location	Tehran University of Medical Sciences, Tehran, Iran
Data accessibility	The data are available with this article
Related research article	Oskoei V, Dehghani M, Nazmara S, Heibati B, Asif M, Tyagi I, et al. Removal of humic acid from aqueous solution using UV/ZnO nano-photocatalysis and adsorption. Journal of Molecular Liquids. 2016;213:374–80.

## Value of the data

• The obtained data present a high efficiency method for removed Acid – 32 – Cyanine 5R dye from the wastewater of textile industries.
• The information of the data includes the methods of zinc oxide nanoparticles plus ultraviolet radiation (UV/ZnO), use of zinc oxide nanoparticles alone (ZnO), and ultraviolet radiation process (UV) in removing Acid – 32 – Cyanine 5R dye from aqueous solutions.
• The research data indicate that the photocatalytic process with the help of ZnO nanoparticles under ultraviolet radiation (UV/ZnO) can completely remove Acid – 32 – Cyanine 5R acidic dye within a shorter time.
• The data will be useful for removing Acid – 32 – Cyanine 5R dye from water and wastewater.

## 1 Data

**Fig. 1:**
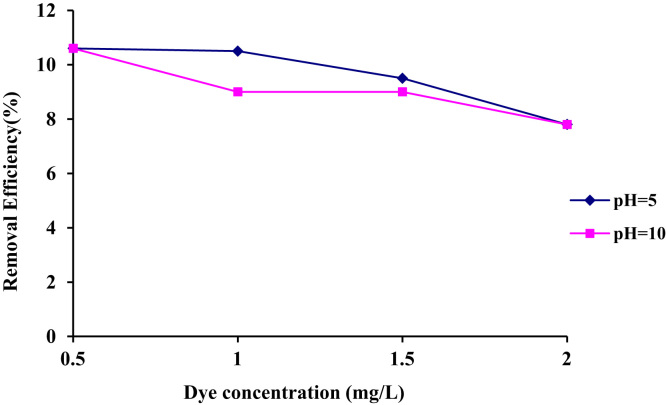
The Acid – 32 dye removal efficiency using ZnO nanoparticles alone under ZnO = 0.2 g/L and contact time of 20 min.

**Fig. 2:**
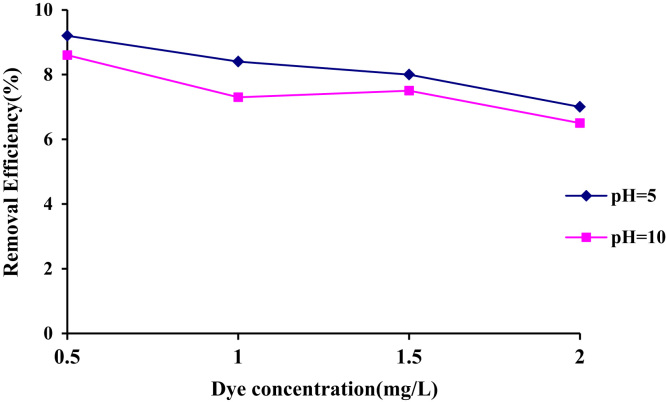
The Acid – 32 dye removal efficiency using ZnO nanoparticles alone under ZnO = 0.1 g/L and contact time of 20 min.

**Fig. 3:**
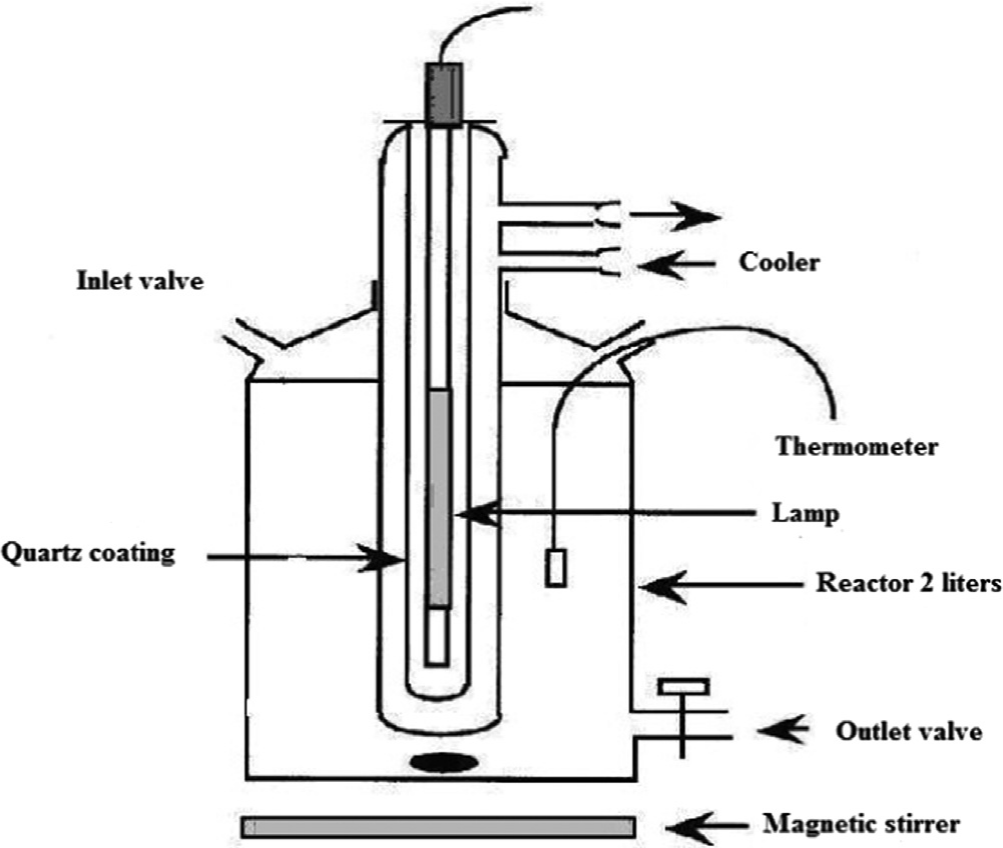
A schematic of the photocatalyst reactor.

**Fig. 4:**
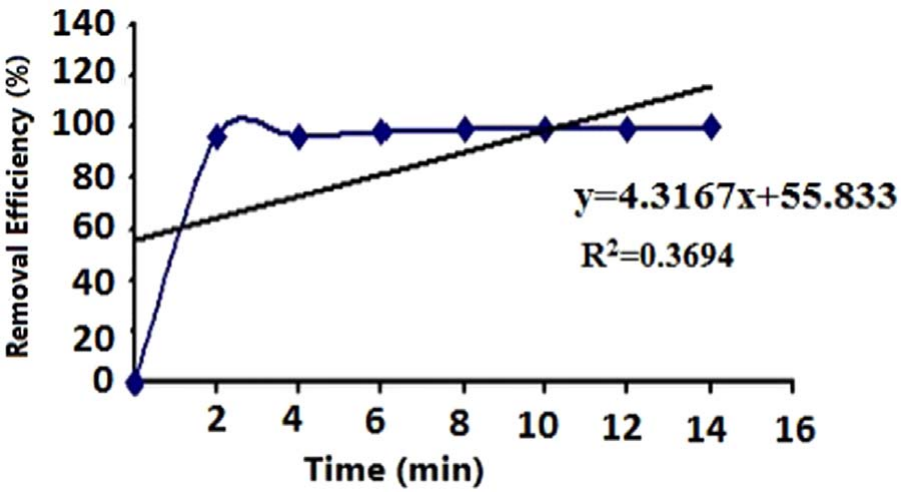
The Acid – 32- Cyanine 5R dye removal efficiency from real wastewater (dyeing) using the nanophotocatalytic method UV/ZnO under optimal process conditions.

The efficiency of Acid – 32 dye removal through radiation with the help of ultraviolet lamp 150 W at different pH has been shown in Tables 1 and 2. Figs. 1 and 2 demonstrate the Acid – 32 dye removal efficiency using ZnO nanoparticles alone within the contact time of 20 min with ZnO dosage of 0.1 and 0.2 g/L. The Acid – 32 dye removal efficiency using the nanophotocatalytic method of UV/ZnO at different pH (5 and 10) and ZnO dosage (0.1 and 0.2 g/L) can be observed. Fig. 3 displays the schematic of the photocatalyst reactor. The qualitative properties of the dyeing wastewater studied here can be seen in Table 7. Fig. 4 reveals Acid – 32 – Cyanine 5 dye removal efficiency from real wastewater (dyeing) using UV/ZnO nanophotocatalytic method under optimal process conditions (Table 3, Table 4, Table 5, Table 6).

**Table 1:** The Acid – 32 dye removal efficiency through radiation by ultraviolet lamp 150 W at pH = 10.

Time (min)	Dye concentration (mg/L)			
	0.5	1	1.5	2
2	67	75	48.5	58.5
4	75	81	53	62.5
6	80	87	60	66.5
8	88	92	65	71
10	90	93.5	70	74.5
12	93	95	75	78.5
14	95	97	79.5	83.5
16	96	97.5	83	86
18	98	98	85	87
20	98.5	98.5	89	89.5

**Table 2:** The Acid – 32 dye removal efficiency through radiation by ultraviolet lamp 150 W at pH = 5.

Time (min)	Dye concentration (mg/L)			
	0.5	1	1.5	2
2	71.5	78	5	59.5
4	79	84	58	64
6	81.5	88.5	63	68.5
8	87.5	93	67.5	73
10	90	94.5	72	76.5
12	93	95.5	79	84
14	94.5	97	83	87
16	96.5	98	86.5	89
18	98	98.2	88	90
20	98.5	99	91	91.5

**Table 3:** The Acid – 32 dye removal efficiency using the nanophotocatalytic method of UV/ZnO at pH = 10 and ZnO concentration = 0.2 g/L.

Time (min)	Dye concentration (mg/L)			
	0.5	1	1.5	2
2	96.6	97	97	97
4	98	97.5	97.5	98
6	98.6	98.2	98.2	98.4
8	100	98.5	99	98.8
10		99.2	99.5	99.4
12		100	100	99.4
14				100

**Table 4:** The Acid – 32 dye removal efficiency using the nanophotocatalytic method of UV/ZnO at pH = 5 and ZnO concentration = 0.2 g/L.

Time (min)	Dye concentration (mg/L)			
	0.5	1	1.5	2
2	96.6	96.4	96.7	97.6
4	97.3	97	97.5	97.6
6	98	97.8	98.2	98.4
8	98.6	98.5	98.7	99
10	99.3	99.2	99.2	99.4
12	100	100	99.2	99.6
14			100	100

**Table 5:** The Acid – 32 dye removal efficiency using the nanophotocatalytic method of UV/ZnO at pH = 10 and ZnO concentration = 0.1 g/L.

Time (min)	Dye concentration (mg/L)			
	0.5	1	1.5	2
2	95.3	95.7	96	96.2
4	96.6	96.8	96.7	97
6	98	97.5	97.7	97.8
8	98.6	98.2	98.2	98.4
10	100	98.5	99	98.8
12		99.2	99.2	99.2
14		100	99.5	99.6
16			100	100

**Table 6:** The Acid – 32 dye removal efficiency using the nanophotocatalytic method of UV/ZnO at pH = 5 and ZnO concentration = 0.1 g/L.

Time (min)	Dye concentration (mg/L)			
	0.5	1	1.5	2
2	94	95	96	96
4	96	96.4	96.7	96.6
6	96.6	97	97.7	97.4
8	98.6	97.8	98.2	98
10	100	98.5	99	98.4
12		98.9	99.2	98.8
14		99.2	99.5	99.4
16		100	100	99.6
18				100

**Table 7:** The qualitative properties of the studied dyeing wastewater.

Parameters	Value
pH	7.5
TSS	205 mg/L
COD	147 mg/L
BOD5	125 mg/L
Dye concentration	385 ADMI

ADMI: American Dye Manufactures Institute.

## 2 Designing the experiments, materials and methods

### 2.1 Materials

All of the chemical used in the data such as Acid – 32 – Cyanine 5R dye, ZnO nanoparticles (purity degree = 99.8%), ultraviolet lamp (150 W), HCl and NaOH were all analytical grade. Stock solution 50 mg/L of Acid – 32 – Cyanine 5R dye was used to prepare the dye samples with the concentrations of interest. ZnO nanoparticles were prepared from NanoPars Spadana Co. [1], [2]. Then, using a furnace at 400 °C, it was activated for 30 min and utilized to perform the dye removal experiments.

The pH of the solutions was measured using a pH meter E 250. All experiments related to COD-BOD_5_-pH variables and TSS were performed according to the standard edition of the 22st ed [3]. The color concentration was determined using the Perkin–Elmer lambada 25-UV/Vis and the true color was measured in ADMI (American Dye Manufactures Institute) units at 400–700 nm wavelengths.

### 2.2 Designing the experiments

All of the dye removal experiments were performed in a glass container with a volume of 2 L and useful volume of 1 L as batch using ZnO nanoparticles as mixed with a dye-containing synthetic solution. After adding the ZnO nanoparticles, the intended solutions were placed on a magnetic stirrer for 20 min in darkness for mixing to occur (IkA-Werke) [1], [2], [4], [5], [6]. After that, the intended solution was transferred to a photocatalytic reactor. Inside the reactor, medium-pressure UV lamp (150 W) was used as the radiation source (Fig. 3). To cool the lamp down and keep the temperature uniform throughout the experiments, a 2-L beaker was placed inside a glass container containing cool water and thermometer, so that the temperature would remain constant around 24 °C across all of the experiments. The pH of the samples of interest was adjusted using HCl 0.1 M and NaOH 0.1 M solutions by a pH meter (pH-meter E 250) [7], [8], [9], [10]. The effect of the optimization parameters including the initial pH (5 and 10), dose of ZnO nanoparticles (0.1 and 0.2 mg/L), contact time (2–20 min), and initial dye concentration (0.5–2 mg/L) was examined on Acid – 32- Cyanine 5R dye removal. The Acid – 32 – Cyanine 5R dye removal efficiency was investigated under the processes of 1) nanophotocatalytic UV/ZnO, 2) radiation alone (UV), and 3) ZnO nanoparticles alone. At the end of the experiments, ZnO nanoparticles were separated from the solution through centrifugation (Hettich – Universal) at 10,000 rpm for 10 min in two stages. After that, the residual concentration of Acid – 32 – Cyanine 5R dye was determined by a spectrophotometer device (Perkin–Elmer Lambada 25 – UV/vis) at the wavelength of 400–700 nm [1], [2], [3], [4], [5], [6].

### 2.3 Investigating the Acid – 32 – Cyanine 5R dye removal efficiency in real dyeing wastewater samples

To evaluate the Acid – 32 – Cyanine 5R dye removal efficiency, samples were taken from the output wastewater of a dyeing workshop. They were then kept at certain time intervals under optimal operational conditions of the reactor. After complete centrifugation of the samples, the maximum absorption of the samples was read by a spectrophotometer, and the extent of reduction of the wastewater dye by the nanophotocatalytic process was determined. Table 7 provides the qualitative properties of the studied dyeing wastewater.

### 2.4 Data analysis

The Acid – 32 – Cyanine 5R dye removal efficiency was determined by the following equation:

(1) $$\mathrm{RE}=\frac{(C_{i}-C_{t})}{C_{i}}\times 100$$

In this equation, *C_i_* represents the initial concentration of the dye in the suspension (mg/L) and *C_t_* shows the final concentration of the dye in the suspension (mg/L) [1], [2].
